# Qualitative study of user perspectives and experiences of digital inhaler technology

**DOI:** 10.1038/s41533-022-00320-9

**Published:** 2022-12-22

**Authors:** Ireti Adejumo, Mitesh Patel, Tricia M. McKeever, Dominick E. Shaw, Manpreet Bains

**Affiliations:** 1 NIHR Nottingham Biomedical Research Centre, Respiratory Medicine, Nottingham, UK; 2 University Hospitals Plymouth NHS Trust, Plymouth, UK; 3 Faculty of Medicine and Health Sciences, University of Nottingham, Nottingham, UK

**Keywords:** Translational research, Asthma, Health care

## Abstract

Electronic monitoring devices (EMDs) have been trialled in interventions to improve inhaled corticosteroid adherence and clinical outcomes. This study sought to understand the perceptions and experiences of EMD end-users. Participants recruited into a six-month EMD study were invited to a semi-structured interview. Interviews were audio-recorded, transcribed verbatim and analysed using the framework approach. Twenty-eight participants (68% female, median age 47) were interviewed. Individuals described feeling responsible for their asthma control. Recent attacks motivated a desire to maintain control. Study participation led to increased awareness of asthma status and medication use. Several individuals were open to integrating digital monitoring data with other mHealth inputs, perceiving the potential to enhance communication with clinicians and empower self-management. Openness to data sharing was tied to expectations of transparent data use. Data supported integrating beliefs and habit formation to achieve behaviour change. There was a willingness for an integrated, platform-based approach to digital self-management.

## Introduction

Asthma is a chronic condition characterised by symptoms of wheeze, breathlessness, chest tightness and cough^1^. Inhaled corticosteroid (ICS) therapy has been the mainstay of treatment for many years and its effectiveness is undisputed^2^. However, asthma is increasingly recognised to be a heterogeneous condition with multiple factors affecting its control^3^. This is leading to a shift towards assessing and treating factors which mediate asthma risk at an individual level. In what has been labelled a ‘treatable traits’ approach, targeted biological interventions are advocated alongside the management of psychosocial and behavioural risk factors^3–5^. Given its prevalence and association with poor outcomes in asthma^6^, poor ICS adherence is widely considered to be a ‘treatable trait’^3,4^.

Electronic monitoring devices (EMDs) are digital devices which date and time stamp medication events. Examples of such events include the opening of a pill-box^7,8^ and the actuation of an inhaler^6,9^. Modern devices upload these data to servers in real-time, offering immediate feedback^9,10^. In asthma trials, their use demonstrates good evidence for improving adherence, but less consistent evidence for improving outcomes^9,11^. Potential reasons include study designs not powered for clinical outcomes and the selection of participants with lower asthma risk^11^.

Theories of behavioural change may assist in identifying targets and mechanisms for action, including aiding understanding why interventions succeed or fail^12,13^. In their systematic review, Holmes et al. found that elements associated with the self-regulatory perspective (self-efficacy, necessity beliefs and concerns about medication) were consistently significantly associated with adherence^14^. This framework has been used in examining adherence behaviours in asthma. Horne et al. found poor self-reported adherence was independently associated with doubts about preventer inhaler necessity and concerns about using preventer inhalers (a necessity-concerns framework)^15^. Another study found that electronically monitored adherence was related to treatment beliefs in keeping with a necessity-concerns framework, motivation to adhere, illness characterisation, community support and routines^16^. An emerging habit-formation literature suggests that using automaticity to overcome the constant requirement for motivation may help newly-formed behaviours to persist^17^.

A pilot study was designed to assess the impact of an EMD-based adherence intervention on treatment decisions and asthma control in a population with increased asthma risk^18^. A qualitative study was conducted as part of the pilot. This aimed to understand study participants’ baseline beliefs and the acceptability of using EMDs over the six-month study period. There is a well-documented disparity between clinicians and their patients on the importance of adherence^19^, as well as evidence of poor real-world engagement with adherence technologies despite relative acceptability^20,21^. Given this, it was of particular interest how this potential target group for digital interventions saw the future role of such interventions in their own routine asthma care.

## Methods

### Pilot study

This qualitative study was nested within a mixed methods pilot randomised controlled trial (RCT)^18^. The pilot study was conducted to assess the feasibility of an EMD-based feedback intervention for improving adherence and asthma control. It is registered on clinicaltrials.gov (NCT02977078) and in the ISRCTN registry (ISRCTN90986892). Inclusion criteria specified a recent exacerbation within the preceding 12 months to enrich the sample for higher risk asthma. All participants used combination inhalers (ICS with long-acting beta agonist, ICS/LABA) to fit the EMD devices used.

Participants were enrolled for six months between December 2016 and December 2018. On their first visit, participants were issued with an EMD (Smartinhaler™, rebranded as Hailie™) and informed in vague terms that it would collect “patterns of inhaler use”.

Intervention participants received feedback on their adherence from a mobile phone application (app) in the form of a dashboard showing the proportion of expected actuations that had been recorded. In addition, the preceding month’s data was reviewed by the study team (IA) and discussed with participants at Visits 2–6. Where there was evidence of SABA overuse or ICS underuse, data were also fed back to the participant’s clinical team with the suggestion to consider whether alterations to management were required.

Control group participants saw a limited version of the mobile phone app which showed a cartoon of their device, its battery and Bluetooth™ pairing status, and the name of the inhaler it was linked to. Data were not fed back to usual care teams. At the final visit, the option to have a summary of their inhaler use data was provided to control participants.

Purposive sampling was intended to achieve a sample of 20–30 participants. This anticipated sample size aimed to achieve theoretical saturation based on a sampling approach by including both adherent and non-adherent participants, the outcome of interest. The Medication Adherence Report Scale for Asthma (MARS-A)^22^ was to be used to inform sample selection during the study and participants were asked to complete this instrument during their final visit. It uses a cut-off of 4.5 (out of a possible maximum score of 5) to indicate adherence. Due to under-recruitment, however, all pilot study participants were invited to take part in the semi-structured interview.

Quantitative data were analysed using STATA v16, StataCorp LLC (Texas). Means and standard deviations (SD) were used for parametric data and medians and inter-quartile ranges (IQR) for non-parametric data. Daily ICS adherence was also reported using mean (SD) in line with the literature. The student’s t-test and Wilcoxon’s Rank Sum were applied to parametric and non-parametric continuous outcomes respectively. Tests of significance were two-sided. This analysis is also described elsewhere^18^.

Ethics approval for the interviews was granted as part of the pilot study by London Central Research Ethics Committee. Participants provided written consent to take part in the qualitative study.

### Semi-structured interview

Interviews were conducted as part of the final study visit between July 2017 and June 2019 by a single investigator (IA) with written informed consent. Participants consented to interviews being audio-recorded. Audio files were transcribed verbatim by an external transcription service.

The semi-structured interview format allowed for exploration of core subjects whilst permitting freedom to explore unanticipated ideas and add clarification where needed^23,24^. General topics included baseline asthma control, medication beliefs, experience with their EMD, perceived impact of monitoring, experience of feedback, and potential future avenues for data capture, application and delivery of an EMD-based intervention in routine care. The interview guide is included in the online supplement (Supplementary Table 1).

### Qualitative data analysis

The framework approach was used for analysis^25^. Familiarisation was conducted by listening to the first three interviews during an initial hand-coding process (IA). Developed codes were re-applied to transcripts and these were then mapped onto A3 sheets. The process was repeated for the next three interviews using themes interpreted from the initial mapping process, allowing for both expansion and refinement. Investigator triangulation was performed by a second investigator (MB) independently examining these transcripts and checking resultant codes for similarity and applicability, ensuring that interpreted themes were grounded in the data. At the end of this process, an initial framework was constructed. Themes were indexed and sorted using NVivo versions 11 and 12 (QSR International)^25^.

A further selection of interview transcripts were coded based on the framework, with ongoing iterative development and refinement to reflect accumulating data. At the end of this, a repeat process of triangulation was performed. The six initial transcripts were also reviewed to ensure that the framework was still relevant. During this process, major codes were extracted from NVivo and circulated to the wider team for input (DES, TMM). The remainder of the transcripts were analysed according to the thematic framework with further theme refinement taking place. Themes were reviewed by two investigators (IA, MB) and finalised by consensus.

It has been argued that additional data can often add greater richness and depth to themes beyond what is traditionally considered the ‘point’ of saturation^26^. Thematic saturation was achieved to the level where additional interviews did not generate new themes or sub-themes but did add depth to the themes that had been interpreted.

### Paradigm and reflexive statement

Constructivist paradigms recognise that participants are not passive objects providing data for analysis but significant actors in the process of creating new knowledge. Similarly, investigators are not neutral when creating new knowledge. This has been described as leading to ‘*co-created findings’*^27–29^. In the context of medical research in particular, this can prove invaluable in helping to *‘fill in the gaps between theory and practice’*^28,30^. Openness of the investigator regarding their role in the knowledge-creation process is essential. One way of doing this is through reflection, and a reflexive statement is offered in the next paragraph for this purpose.

Interpersonal interactions and preconceptions coloured how both the interviewer (IA) and participants projected themselves. The initial aim to minimise the investigator’s voice in the transcript led to a central role for non-verbal communication (it was explained to participants that these cues would not denote approval or disapproval)^24^. Some internal tension arose from this investigator’s (IA) medical background and a subsequent felt need to be an educator, requiring significant discipline to simply enquire and listen. Further challenges included avoiding jargon (e.g. “preventer” and “reliever” inhalers) which formed a language barrier, and managing the power dynamic^24,31^.

### Participant identification and reporting standards

Quotes are included in the text. Participants are identified by their study group (control or intervention), their sex and their age. The Standards for Reporting Qualitative Research (SRQR) were adopted^32^.

### Reporting summary

Further information on research design is available in the Nature Research Reporting Summary linked to this article.

## Results

### Participants

**Fig. 1:**
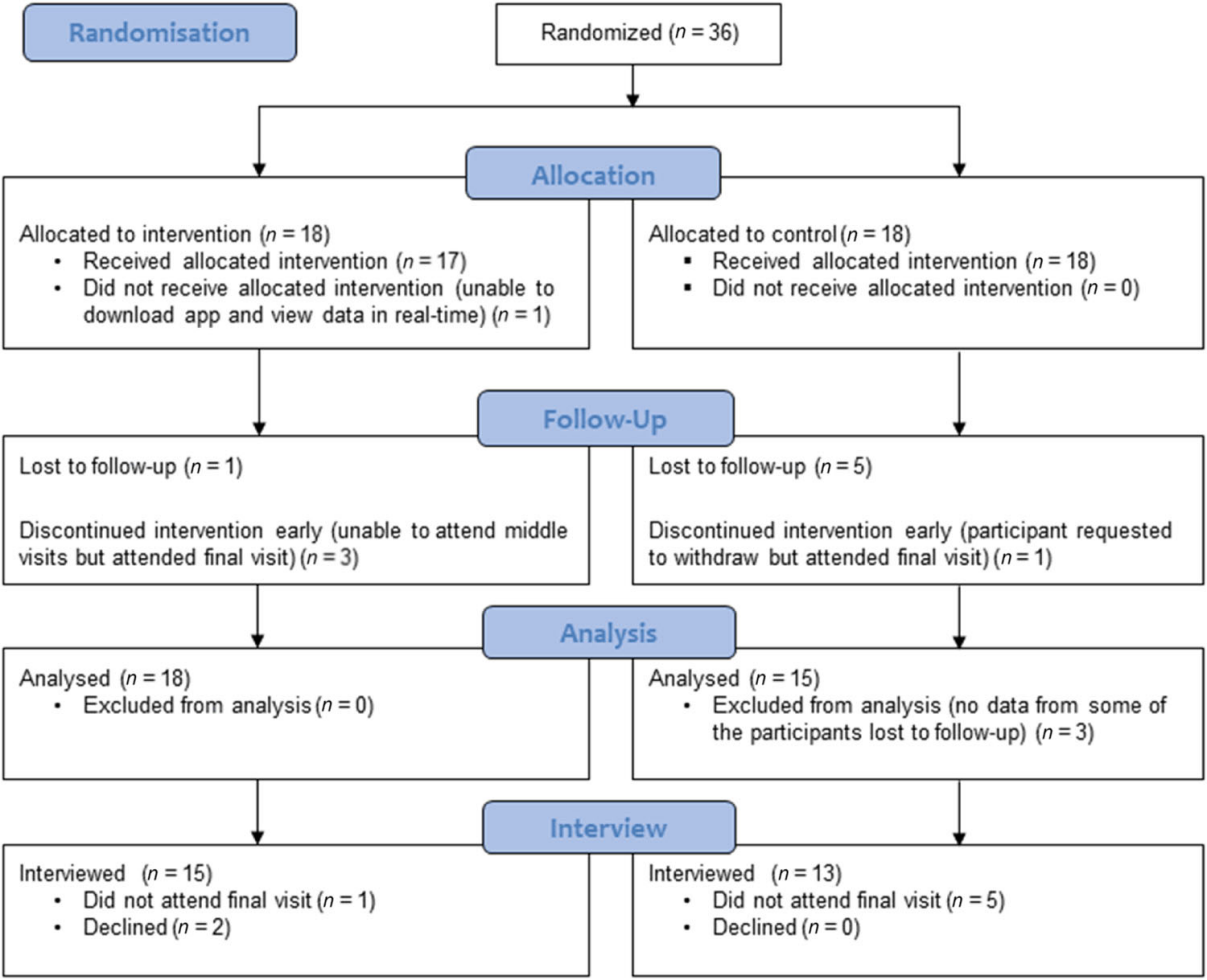
Flow diagram. Flow diagram for interview recruitment where *n* represents the number of participants.

A diagram detailing study flow for the 36 recruited pilot study participants is shown in Fig. 1.

Twenty-eight participants consented to the semi-structured interview (Fig. 1, Table 1). There were no marked differences in age or sex between participants who provided an interview and those who did not. Interviewees had a non-significant greater median adherence (p = 0.534) than non-interviewees (Table 1).

**Table 1:** Participant demographics and adherence.

	Non-interview participants	Interview participants	Control participants	Intervention participants
Number	8	28	13	15
Median age (IQR)	50.4 (33.2, 57.0)	46.7 (33.5, 54.2)	41.9 (32.2, 49.5)	50.3 (34.9, 58.6)
Female n (%)	5 (63)	19 (68)	8 (62)	11 (73)
Caucasian n (%)	6 (75)	24 (86)	10 (77)	14 (93)
Percentage adherence, mean (SD)^a^	65.9 (32.2)	65.5 (32.6)	60.2 (34.4)	70.1 (31.4)
Percentage adherence, median (IQR)^a^	59.3 (49.3, 96.5)	81.0 (36.5, 91.5)	53.4 (36.3, 88.2)	83.4 (43.6, 96.3)

^a^Three participants were lost to follow-up and provided no electronic data and are therefore excluded from this analysis, therefore in the non-interview group only, *n* = 5.

The MARS-A instrument was used to assess adherence in 25 participants. Eleven participants achieved a score of 4.5, indicating that the sample included both individuals who were adherent and non-adherent to their treatment. This is supported by the objective EMD data which indicates a range of ICS adherence during the study period from 4.5% to overuse at 111.0%. Overall results for the study have been published elsewhere^18^.

### Summary of themes

Five themes relating to participants’ experiences prior to and during the study, and their perceptions of applications beyond the study were interpreted (Table 2). The themes and sub-themes are included in full in the supplement (Supplementary Table 2).

**Table 2:** Themes.

Themes	Theme headings
Theme 1:	Participants’ experiences of asthma
Theme 2:	Participants’ experiences of asthma treatment
Theme 3:	Participants’ experiences of study participation and EMD use
Theme 4:	Future applications of digital inhaler technology—potential improvements and uses
Theme 5:	Future applications of digital inhaler technology—desirability, ethics and wider impact

### Participants’ experiences of asthma and its treatment—the context for EMD use

Participants described recent exacerbations as eye-opening, motivating a desire to maintain/regain asthma control. Poor symptom control was generally defined as reliance on reliever inhalers or recurrent/persistent symptoms. It was described as causing limitation and frustration.

Generally, participants felt in control of and responsible for their asthma; although given the relatively high level of overall adherence, this may reflect the study sample. One young participant who did not fit this description explained the following:

“*I feel like the doctors should have responsibility over my asthma but I don’t feel like they do right now… So I do feel like I’m the only one responsible for my asthma at the minute because I’m just not getting any help from my GP.” Female intervention participant, aged 18 years*.

The same participant expressed a lack of belief in the efficacy of their current ICS. This combination of an attenuated sense of self-efficacy and drug efficacy was consistent with her poor adherence despite being in the intervention group (17% doses taken as prescribed)^14,15,33^, highlighting that EMD provision does not necessarily modify underlying beliefs.

Perceptions of inhaler efficacy were almost always tied to previous experiences of symptom burden during inhaler use. Beliefs about necessity were similarly in the context of previous experiences; but also related to fear/lack of fear with regards to what could happen if their ICS was stopped.

Participants described habit as a key to adherence. Habit-forming cues included visual prompts, other household members, written reminders and alarms (although some found these intrusive). Busyness and shift work, transition from adolescence to adulthood (possibly due to increased requirement for self-efficacy) and perceived lack of treatment effectiveness made adherence more difficult.

### Participants’ experiences of the electronic monitoring device

Most participants found the devices bulky. This was generally seen as a negative characteristic, associated with decreased ease of use or increased social embarrassment. A few participants volunteered frustration where data was felt to be unreliable, leading to loss of trust and disengagement.

Participants were generally aware that their inhaler use was being monitored and were broadly accepting of this. A few described being suspicious of data collection as a principle, and concerned about their poor adherence being visible. However, they also accepted monitoring that would benefit themselves or others with asthma.

*“…it’s a little bit sort of why are you keeping tabs on me, but then also it’s good because then if you… notice anything that is a little bit untoward maybe you’re getting the help sooner than not.” Female intervention participant, aged 34 years*.

Both control and intervention participants noted that the awareness of being monitored increased their awareness of how they used their inhalers, including implications for current asthma status.

“*… If I did forget to take it, I thought ‘now that’s going to ruin my… percentage and things…’” Female intervention participant, aged 58 years*.

“*I did like to use the app, especially the Ventolin, sometimes quite shocking actually because sometimes I’d had 10 doses in a 24* *hour period or even more, and I hadn’t realised that I’d done it that many times.” Female intervention participant, aged 58 years*.

Some participants found that their awareness of being monitored reduced with time. There was some suggestion from pilot study data that this awareness may have been partially maintained by feedback; with the intervention group demonstrating greater adherence in the middle months of the study, although this finding was not significant (*p* = 0.29)^18^.

Access to objective inhaler use data gave a few participants more confidence to request support from their clinical teams. Not many clinical teams were reported as having engaged with the data. This was particularly an issue where a participant had no clear options for treatment escalation.

### Future applications of digital inhaler technology—potential improvements and uses

There was consensus that embedding EMD technology within inhalers would be superior to attached devices, although the financial and environmental costs of this were questioned.

When discussing the contexts for feedback of EMD data in routine care, most participants felt feedback should be coordinated by primary care given pre-existing relationships, access to health records, and ability to provide continuity of care.

“*…because I’ve used this particular doctor’s surgery on and off since I was 11, they kind of know you… So, you’re more comfortable with them and you’re more happy, I think, to talk about how you’re feeling…” Female control participant, aged 44 years*.

Many participants accepted elements of remote feedback, but few wanted it to replace in-person interactions altogether. Most expected clinician feedback to be situated in the context of their routine asthma reviews.

Participants primarily anticipated that EMD data would provide their healthcare teams with detailed, accurate and objective information to inform better decision-making in the future. Most expressed a desire for joint responsibility to self-monitor and expected support in interpreting data in a way that would have meaning and utility.

Several participants felt that platforms integrating inhaler use data with environmental (e.g. pollen counts, pollution), physiological (e.g. heart rate, lung function) and activity (e.g. step counters and night-time wakening) data could be useful. Anticipated uses included tracking associations with reliever use, providing objective markers of disease severity and automated self-management advice.

“*… if you could track somebody’s movements all round, you could see where they’ve been, what the weather was like and you’d probably understand the symptoms of asthma better …” Male control participant, aged 57 years*.

Many participants accepted the use of global positioning system (GPS) data to personalise such information, with several noting existing routine use of such data. Some felt GPS data use should be conditional on guarantees of data security, ethical use and the ability to opt-out. For a few, GPS data use was *“invasive”* and *“irrelevant”*, highlighting concerns about privacy and autonomy.

“*…on my phone I tend to turn my location off unless I really need it for maps because I don’t like the idea of people always logging on and seeing where I am, so I think that is quite a big privacy issue.” Female control participant, aged 22 years*.

Autonomy also appeared to be a concern when discussing automated responses to acute deterioration. Several participants communicated that they did not want to lose control of decision-making, even if acutely unwell. This need for control may have been why phone calls and in-app alerts were seen as more acceptable than paramedics responding in person to automated alerts.

### Future applications of digital inhaler technology—desirability, ethics and wider impact

Overall, participants were open to having an EMD in the future, largely based on the potential to facilitate better communication with clinical teams, empower self-management, and reduce health system burdens. There were concerns around depersonalisation, highlighting that a fully remote, automated system was unlikely to be acceptable as part of routine care.

“*… there’s always a risk of becoming slightly more anonymous as a patient I guess, if there’s more, if someone relies more on just data.” Female intervention participant, aged 54 years*.

There was a general feeling that in the current digital age, data collection was neither new nor unnerving, although some expressed concerns about location and audio-visual data collection. Most felt strongly that the data would need to be stored within National Health Service (NHS) systems to ensure security, data integrity and integration with medical health records to facilitate continuity of care. Some were happy for data to be stored on manufacturers’ servers on the condition that access was regulated, that data remained anonymised, and that there was NHS oversight.

Participants were open to data being used for wider research and service planning. There were mixed opinions on who should or should not have access to data, with concerns expressed around pharmaceutical companies, health insurance companies and government.

“*I think if they’re going to make them rich, somebody you can’t trust, I’m not against rich people but do you understand what I mean?! … I’m doing this study so that things can get better, not for companies to make millions of pounds, if you see what I mean.” Male control participant, aged 57 years*.

Others felt that access to outside parties was important, for ongoing product development for example. Several participants made it clear that access should be on a need-to-know basis, that they would want to know who had access to their data, what it was being used for, and retain the right to opt out of data sharing. This was seen as a matter of trust.

“*I suppose it doesn’t matter who has access, so long as you’re clear to whoever you’re capturing that data from, that that’s where it’s going, I think that’s actually more important than who should or shouldn’t, I think so long as you’re informed and you have that choice as to whether you want that particular company or person to have access to that data, that’s probably more important.” Male control participant, aged 42 years*.

## Discussion

Participants in an EMD intervention pilot study described the importance of past experiences to current health beliefs and a sense of responsibility to achieve and maintain symptom control. Participants described increased awareness of their condition, in part attributed to EMD data. They were generally open to integrating EMD data with environmental, physiological and activity data, and saw ways in which such technologies could enhance asthma care.

Similar to findings in this study, previous investigators have noted heterogeneous responses to EMD use. Some adolescents have described feeling an increased sense of control and responsibility for their condition^34^. Others saw the monitoring as a sign their clinicians did not trust them^35^. Also similar are previous studies where adults and adolescents described behaviour and/or attitude change from the using EMDs (including *“habit formation”*), with some adults linking this to better asthma control^34,36^. Perceptions of how durable these changes would be were mixed^35,36^. These changes appeared to be somewhat linked to baseline attitudes and beliefs, and pre-existing adherence to or dislike of routines^36^.

Other studies have also described data facilitating conversations with healthcare teams^34,36^, a desire for data access to be limited^34^ and concerns that data would replace them being seen as a person^35^. Although reminders were not formally used in this study, some participants reported finding and using this function. The literature suggests variable acceptance of reminder functions with potential implications for their use^34–36^.

The association between a self-regulatory perspective (including its specific application, the necessity-concerns framework) and adherence in asthma has already been outlined. Participants discussed beliefs about asthma and its treatment, influenced by their varying experiences, that appeared to motivate or demotivate inhaler use in keeping with what is already known^15,36,37^. As has also been previously noted, there were elements in addition to the necessity-concerns framework which appeared to play a role^16^.

Participants in this study described being motivated to avoid recurrence of the kind of deterioration that had led to their recent exacerbations, highlighting a target population that could benefit from intervention. In a Protection-Motivation model of behaviour change, a ‘*threat appraisal’* of susceptibility, severity, and fear are central to motivating intention to take on adaptive behaviours to address the threat^38^.

Importantly, Protection Motivation Theory notes a risk of maladaptive responses, including avoidance, denial and hopelessness, where an accurate *threat appraisal* has been made but there is low belief in treatment efficacy, high concern about treatment costs and low self-efficacy^38^. This may in part explain why some intervention participants demonstrated persistently poor adherence and why other narratives around poor ICS efficacy and low self-efficacy appeared to blunt intentions to adhere to treatment.

Previous work has similarly identified a subgroup of individuals with poor adherence which is resistant to intervention^39–41^. EMD-based interventions may aid identification of this group, facilitating sensitive exploration of underlying beliefs and adaptation of interventions. This could prove key for some in tipping the balance in favour of adherence-concordant beliefs and adaptive behaviours^38,42,43^.

Participants also highlighted the importance of cues which were visual, auditory and events-based, a finding also seen by Foster et al. in their study^36^. Participants perceived that factors which disrupt routines (e.g. shift work) negatively impacted their adherence. Habit theory proposes that, whilst beliefs inform initial motivation to begin a new behaviour, habit formation embeds behaviour change by rendering a new behaviour automatic. This allows new behaviours to be maintained long after both motivation and awareness have ended. For such automaticity to take place, a new behaviour must be learned in an enabling environment, a critical cue for action identified and a plan put in place to perform the desired action when cued^44^.

Overall, data from this study suggest that, in a selected post-exacerbation population, EMD interventions comprising clinician input have potential to influence beliefs and increase motivation for ICS adherence. They may also assist in identifying individuals who need more complex engagement around treatment efficacy and concerns, and self-efficacy. Finally, they may provide an opportunity to target habit formation as a means of embedding behaviour change.

EMD research in asthma has primarily focused on its potential to reduce adverse risk through improved adherence. Participants in this study however were curious about the potential for an integrated technology platform to inform lifestyle choices such as exercise and trigger avoidance. They wanted to integrate data with environmental data, physiological and activity markers, and validated symptoms to self-monitor and provide better information for shared decision-making with their clinical teams, a finding supported by previous work^19^.

Self-management is central to chronic disease care where clinician input is limited by time. The US Institute of Medicine suggests it comprises *“confidence to deal with medical management, role management and emotional management”* of a condition^45^. In asthma, supported self-management has been shown to be effective in improving outcomes^46,47^. Evidence suggests that self-management behaviours are most effectively influenced when clinicians take the time to engage with individuals^37^.

By providing clinicians with the same tailored information available to users, integrated with markers of modifiable factors and outcomes that matter to users, EMD-based interventions may provide a common language to increase engagement of users with their self-management and of clinicians with their patients. Personalisation has the potential to allow for tailored self-management interventions, including personalised asthma action plans informed by data, automated advice and access to mental health support using validated apps.

Ethical data use is key, with an expectation of data security, transparency over what data are being used for and of some level of control over data access. Without this, there is a risk of trust breaking down. This is particularly key when considering the implications of platform technologies that allow for the integration of commercial sensor data with potentially sensitive health data, and where development of automated interpretation is likely to involve algorithms which require training using existing data. Recent controversies highlight the importance of transparency^48,49^ in such circumstances.

Participants in this study were largely keen for EMD-based interventions to be delivered in primary care, enhancing rather than replacing their routine reviews. Given that EMDs’ key role is likely to be in supported self-management, this appears a natural choice. However, primary care services in many healthcare systems are already under pressure, meaning that careful thought is needed for implementation. This study suggests that, without training or allocated interpretation time, clinicians generally did not find EMD data helpful in informing management.

For monitoring interventions to be successful, individuals need assistance in processing and effectively utilising the data gained^50^. This is something that participants from this study actively expected, but will require clinician training and time for them to be able to interpret and use the data. Data outputs will therefore need to be presented in ways that are interpretable for users with asthma, as well as standardised and clinically useful for their clinicians. If data is to be from a platform source, automated integration and interpretation is likely to be required. This will need to add value and reduce clinician burden/time, for example by permitting remote monitoring and passive collection of inputs that form a core part of the asthma review.

In placing a spotlight on the expert perspectives of end-users at high risk of adverse events, this study places those most likely to benefit from digital interventions at its centre. The interview’s timing at final visit maximised participation by limiting inconvenience, also aiming to reduce recall bias. This may, however, have reduced the opportunity for participants to process and contextualise their experiences. Other limitations include the overwhelmingly female and Caucasian sampling. Participants volunteered for the pilot study, potentially self-selecting as a group more likely to be engaged in their self-management. This is likely reflected in the relatively high adherence rates seen. Findings are furthermore the perceptions and experiences of this unique group of individuals, who offer their own valuable insights and perspectives.

In conclusion, data from participants of a pilot interventional study supports model integrating beliefs and habit formation to achieve behaviour change. Participants expressed a willingness for a more integrated, platform-based approach to digital self-management, but were clear that they expected their data to be used ethically. This study finds a general optimism for the potential of inhaler technology to have both personal and wider impacts on self-management and on shared decision-making.

## Supplementary information

Supplementary Information

REPORTING SUMMARY

## Data Availability

Anonymised data will be made available by the corresponding author. This includes (but is not limited to) requests for the purpose of secondary analysis to investigators who provide a methodologically sound proposal.
