# Supplementary material for: Qualitative study of user perspectives and experiences of digital inhaler technology
Source: NPJ Prim Care Respir Med. 2022 Dec 22;32:57. doi: 10.1038/s41533-022-00320-9 (PMC9780314; doi:10.1038/s41533-022-00320-9)
Supplement: Supplementary file 1 — Supplementary Information [file 41533_2022_320_MOESM1_ESM.docx]

Qualitative study of user perspectives and experiences of digital inhaler technology - supplement

Ireti Adejumo^1^, Mitesh Patel^2^, Tricia M. McKeever^1^, Dominick E. Shaw^1^, Manpreet Bains^3^

^1^NIHR Nottingham Biomedical Research Centre

^2^University Hospitals Plymouth NHS Trust

^3^Faculty of Medicine and Health Sciences, University of Nottingham

| Dr Ireti Adejumo  (corresponding author)  Clinical Sciences Building  Division of Respiratory Medicine  City Hospital Campus, University of Nottingham  Hucknall Road  Nottingham  NG5 1PB  United Kingdom  [ireti.adejumo@nottingham.ac.uk](mailto:ireti.adejumo@nottingham.ac.uk)  0115 823 1935 |
| --- |

# Supplement

## Supplementary Table 1: Interview guide

| **Introduction**  Explain the purpose of the interview in general:  *‘We would like to hear how you felt about:*   - *Your participation in the Smartinhaler™ study* - *Your views on the device* - *Your views on the collection of personal electronic data from your Smartinhaler™* - *Your views on the collection of personal electronic data from your mobile phone.’*   Check consent form has been signed and check still happy to take part.   1. Give statement on confidentiality, right to withdraw consent, recording of the interview:   ***‘We would like to reassure you that all data relating to yourself will be kept strictly confidential by the research team. The recording of this interview and any quotes used in study reports will not identify you in any way. Your participation is entirely voluntary and you are free to stop the interview at any time without giving a reason.We will retain any information collected to this point unless you specify otherwise.’***   1. Ask if the participant has any questions before starting the interview. 2. Explain that the interview will last between 30 and 45 minutes. |
| --- |
| **Background**  Discussion of baseline asthma control   1. Tell me about your asthma – how is it normally? – how does it affect you? 2. How long have you had asthma for? How has it been treated over the years? 3. To what extent do you feel you’re in control of your asthma?    1. How often you’re getting symptoms    2. The effect it’s having on your life    3. The number of times you’re needing to see your GP/go to hospital 4. What did you think of your inhalers prior to enrolling in the study?    1. Did you feel you needed them?    2. How effective were they for your asthma?    3. According to your prescription, how regularly was your preventer inhaler meant to be used?    4. How regularly did you use your preventer? Explore.    5. Did you use any prompts to remember to use your preventer (e.g. phone alarms, calendar reminders)?    6. How did you feel about using them publicly?   Exploration of baseline health beliefs   1. How important do you feel it is to take your preventer inhaler regularly?    1. If I made the statement “Regular use of your inhalers as prescribed is **crucial** to preventing your asthma from getting worse or flaring up,” would you find yourself able to agree or would you have to disagree?    2. What makes you feel this way? 2. How important do you feel it is to take your other asthma medication regularly?    1. What makes you feel this way? |
| **Smartinhaler™ Experience**  Discussion opening   1. Can you share your general thoughts on the device you have been using?    1. What did you think of it?    2. How did it compare to using your inhaler without the device?    3. How easy was it to carry around? Is this any different to your normal inhaler?    4. How easy was it to use in public? Is this any different to your normal inhaler?   Exploration of perceived impact on inhaler usage subsequent to being informed of monitoring   1. To what extent do you think your participation in the study impacted the use of your inhaler?    1. Did it impact how regularly you used your preventer?    2. Did it impact how regularly you used your reliever?    3. Do you feel it changed how you used your inhalers in any other way?    4. Do you feel it changed how important you feel taking your preventer regularly is? 2. I asked earlier whether you used any prompts before to remind you to use your inhalers.    1. Did you find you used any prompts during the study?    2. Did you find yourself more or less in need of those prompts during the study?   Discussion of response to feedback   1. Can you tell me about any feedback you received?    1. Through the smartphone app?    2. From your GP/practice nurse?    3. From your hospital clinician?    4. From any other sources? 2. How did the feedback make you feel? 3. How useful was the feedback? 4. How clear was the feedback? 5. Can you suggest any ways in which the provision of the feedback could be improved?   Discussion of effect of participation on other aspects of self-management.   1. To what extent has participation in the study helped with how well your asthma has been controlled?    1. How often you’re getting symptoms    2. The effect it’s having on your life    3. The number of times you’re needing to see your GP/go to hospital 2. To what extent has participation in the study helped with how well *you* have been able to take control of your asthma? 3. Have you learnt more about your asthma from participating in this study?   Exploration of reaction to monitoring   1. What were you told about the device when it was given to you? 2. Were you aware that the way you used the inhaler was being monitored?    1. **If yes**: what do you think was being monitored?    2. What did you think about this? How did it make you feel?    3. If you hadn’t been aware you were being monitored, would that change how you feel now?    4. To what extent did knowing you were being monitored change the way used your inhaler?    5. **If no:** what do you think might have been monitored?    6. Explain monitoring took place    7. Explain what was monitored    8. ‘How do you feel about this?’    9. If you had been aware ……… was being monitored, would that change how you feel now? |
| **Data Capture**   1. If, in the future, we started monitoring inhalers like we did with you, who, in your opinion, should be responsible for keeping an eye on the data?    1. E.g. You? Your GP/practice nurse? The hospital?    2. What makes you feel this way? 2. Where do you feel that healthcare-related data like this should be stored? **(NB these are NOT your medical records)**    1. E.g. on the company’s database? On a GP or hospital database? 3. If, in the future, we started monitoring inhalers like we did with you, who, in your opinion, should be responsible for providing the feedback?    1. E.g. Your GP/practice nurse? The hospital? A dedicated lay service?    2. How should it be provided? (text, email, web link, phone call etc) 4. Who do you feel has the ultimate responsibility for your asthma at the moment?    1. Who should have/would you like to have responsibility for it?    2. Who else might help you in controlling your asthma symptoms? 5. How would you feel about data on how you use your inhaler being linked to data on your asthma triggers (local pollen levels, for example)?    1. How would you feel about the use of location tracking via GPS to increase the accuracy of this?    2. Do you feel there is a possibility this could be used to help with your asthma? 6. How would you feel about linking information from fitness or healthcare apps you already use or might use in the future to give us more information relevant to your asthma? 7. Is there anything else you feel we could collect data on that might help us get a better idea of the factors involved in what makes your asthma easy/hard to control? 8. Is there any type of data capture (information on your asthma that we could collect using technology) that makes you nervous? 9. Who do you think should or shouldn’t have access to this sort of data? |
| **Future applications**   1. Would you consider using a Smartinhaler™ regularly?    1. What are the main reasons you think/don’t think so? 2. How would you feel if your GP or consultant provided you with a Smartinhaler™ permanently/for a short period of time? 3. How do you think your GP or consultant might use the data obtained from it? 4. How would you feel if your GP or consultant/another healthcare professional/a nonhealthcare professional:    1. Discussed the data they had obtained from it with you?    2. Asked you to change something based on this data?    3. Carried out an emergency intervention based on this data?    4. Used it to monitor your response to treatment? 5. How often do you think these discussions should take place? *E.g. monthly, as and when needed* 6. How would you feel if this information was shared with other members of your healthcare team? 7. How would you feel about this data being used to inform what treatment you are/are not prescribed?    1. For example, we may consider using them to tell us if people can start newer medications for asthma in the future. How would you feel about them being used in this way?    2. If Smartinhalers™ were to be used in this way, do you think they would impact the way your GPs interact with you? *E.g. your annual asthma review*    3. If so, how? If not, why?    4. How do you feel about this? |
| **Smartinhaler™ going forward**   1. Can you think of any ways in which the Smartinhaler™ could be designed differently?    1. Is there anything in the design of the device that: 2. Could be improved?    1. Would make it easier to use?    2. Would make you more likely to want to use it?    3. Is there anything in the design of the app that: 3. Could be improved? 4. ii. Would make it easier to use?    1. Would make you more likely to want to use it? 5. How would you feel if there was no casing at all and the Smartinhaler™ was just part of your inhaler? 6. Would you ever consider purchasing a Smartinhaler™ for use yourself? Why/why not? 7. How much do you think the Smartinhaler™ costs?    1. What do you think about the cost?    2. How does this affect whether you would consider purchasing one? 8. Can you think of anything else the Smartinhaler™ might add    1. To your current care?    2. To the way we manage asthma in general? |
| **Closing Remarks**   1. Is there anything that we haven’t talked about that is important to you and that you’d like to add? 2. Do you have any questions for me? 3. Thank the participant for their time. |

## Supplementary Table 2: Themes and sub-themes

| *Themes* | *Subthemes* |
| --- | --- |
| Theme 1: Participants’ experiences of asthma | *Beliefs and attitudes* |
|  | *Participants’ experiences of their asthma symptoms* |
|  | *Participants’ experiences of healthcare services* |
| Theme 2: Participants’ experiences of asthma treatment | *Participants’ beliefs about asthma treatment* |
|  | *Experiences of changes to asthma treatment* |
|  | *Patterns of inhaler use* |
|  | *Participants’ experiences of using their inhalers in public* |
| Theme 3: Participants’ experiences of study participation and EMD use | *General comments about participating in research* |
|  | *Participant experiences of using the Smartinhaler™ system* |
|  | *Participants’ comments on study feedback* |
|  | *Participants’ awareness of being monitored* |
|  | *Acceptability of monitoring to participants* |
|  | *Impact of the study on awareness and control* |
|  | *Impact of the study on behaviour* |
|  | *Participants do not perceive an impact from participating in the study* |
| Theme 4: Future applications of digital inhaler technology – potential improvements and uses | *Future characteristics of a digital inhaler system* |
|  | *Views on the nature of feedback* |
|  | *Views on potential future uses of digital inhalers* |
| Theme 5: Future applications of digital inhaler technology – desirability, ethics and wider impact | *Desirability of the Smartinhaler™ system* |
|  | *The subject of data ethics* |
|  | *Participants’ views on the potential wider impact of* *digital inhaler technology* |
