# Supplementary material for: Qualitative study of user perspectives and experiences of digital inhaler technology
Source: NPJ Prim Care Respir Med. 2022 Dec 22;32:57. doi: 10.1038/s41533-022-00320-9 (PMC9780314; doi:10.1038/s41533-022-00320-9)
Supplement: Supplementary file 2 — REPORTING SUMMARY [file 41533_2022_320_MOESM2_ESM.pdf]

nature portfolio

Corresponding author(s): Ireti Adejumo

Last updated by author(s): Jul 6, 2022

Reporting Summary

Nature Portfolio wishes to improve the reproducibility of the work that we publish. This form provides structure for consistency and transparency in reporting. For further information on Nature Portfolio policies, see our Editorial Policies and the Editorial Policy Checklist.

Statistics

For all statistical analyses, confirm that the following items are present in the figure legend, table legend, main text, or Methods section.

n/a	Confirmed	
☐	☒	The exact sample size (*n*) for each experimental group/condition, given as a discrete number and unit of measurement
☒	☐	A statement on whether measurements were taken from distinct samples or whether the same sample was measured repeatedly
☐	☒	The statistical test(s) used AND whether they are one- or two-sided<br>*Only common tests should be described solely by name; describe more complex techniques in the Methods section.*
☒	☐	A description of all covariates tested
☒	☐	A description of any assumptions or corrections, such as tests of normality and adjustment for multiple comparisons
☐	☒	A full description of the statistical parameters including central tendency (e.g. means) or other basic estimates (e.g. regression coefficient) AND variation (e.g. standard deviation) or associated estimates of uncertainty (e.g. confidence intervals)
☒	☐	For null hypothesis testing, the test statistic (e.g. *F*, *t*, *r*) with confidence intervals, effect sizes, degrees of freedom and *P* value noted<br>*Give P values as exact values whenever suitable.*
☒	☐	For Bayesian analysis, information on the choice of priors and Markov chain Monte Carlo settings
☒	☐	For hierarchical and complex designs, identification of the appropriate level for tests and full reporting of outcomes
☒	☐	Estimates of effect sizes (e.g. Cohen's *d*, Pearson's *r*), indicating how they were calculated

Our web collection on statistics for biologists contains articles on many of the points above.

Software and code

Policy information about availability of computer code

Data collection	n/a
Data analysis	Quantitative data were analysed using STATA v 16, StataCorp LLC (Texas). Qualitative data were organised in NVIVO versions 11 & 12 (QSR International).

For manuscripts utilizing custom algorithms or software that are central to the research but not yet described in published literature, software must be made available to editors and reviewers. We strongly encourage code deposition in a community repository (e.g. GitHub). See the Nature Portfolio guidelines for submitting code & software for further information.

Data

Policy information about availability of data

All manuscripts must include a data availability statement. This statement should provide the following information, where applicable:

- Accession codes, unique identifiers, or web links for publicly available datasets
- A description of any restrictions on data availability
- For clinical datasets or third party data, please ensure that the statement adheres to our policy

Anonymised data will be made available on reasonable request to the corresponding author.

Human research participants

Policy information about studies involving human research participants and Sex and Gender in Research.

Reporting on sex and gender	Findings are reported for both male and female sex. Data on gender was not collected in this study.
Population characteristics	Median age of interview participants was 47 years. 68% of participants were female. 86% of participants were self-identified as Caucasian.
Recruitment	Participants were recruited from primary and secondary care, the Nottingham Respiratory Research Database (NRRD), and by public advertisement. Participants volunteered for the pilot study, potentially self-selecting as a group more likely to be engaged in their self-management; however there is evidence that participants with poorer adherence were included in the study.
Ethics oversight	London Central Research Ethics Committee.

Note that full information on the approval of the study protocol must also be provided in the manuscript.

Field-specific reporting

Please select the one below that is the best fit for your research. If you are not sure, read the appropriate sections before making your selection.

☐ Life sciences ☒ Behavioural & social sciences ☐ Ecological, evolutionary & environmental sciences

For a reference copy of the document with all sections, see nature.com/documents/nr-reporting-summary-flat.pdf

Behavioural & social sciences study design

All studies must disclose on these points even when the disclosure is negative.

Study description	A qualitative study nested within a pilot randomised controlled trial assessing the effect of electronic monitoring devices (EMDs) on asthma control and treatment decisions. The qualitative study aimed to understand participant perceptions of EMDs and potential for use in future care.
Research sample	As per the study objectives, individuals were invited to participate in the pilot study if they had doctor-diagnosed asthma for at least 12 months, were using an inhaler compatible with the electronic monitoring devices used and had self-reported exacerbation in the preceding 12 months. Participants were required to be aged 18-65 as this was an adult study targeting asthma rather than COPD. All participants from the pilot study undertaking the final visit were invited to participate in the qualitative study.
Sampling strategy	No sample size calculation was made. From the literature, it was anticipated that a sample of 20-30 participants would provide adequate depth and saturation. Given the numbers recruited to the pilot study, a convenience sample with all participants attending their final visit as the pool was adequate to achieve this.
Data collection	Interviews were conducted by one researcher and audio-recorded. These were transcribed by an external transcription company with transcripts anonymised, identified by only the study number. Data collection was not blinded.
Timing	Interviews were conducted as part of the final study visit between July 2017 and June 2019.
Data exclusions	All participant interviews were included in the analysis.
Non-participation	Of 36 participants recruited to the pilot study, 6 did not attend the final visit. Of the 30 participants who did attend the final visit, 2 declined to participate in the interview.
Randomization	Participants were randomised to study groups using computer-generated codes.

Reporting for specific materials, systems and methods

We require information from authors about some types of materials, experimental systems and methods used in many studies. Here, indicate whether each material, system or method listed is relevant to your study. If you are not sure if a list item applies to your research, read the appropriate section before selecting a response.

Materials & experimental systems

n/a	Involved in the study
☒	☐ Antibodies
☒	☐ Eukaryotic cell lines
☒	☐ Palaeontology and archaeology
☒	☐ Animals and other organisms
☐	☒ Clinical data
☒	☐ Dual use research of concern

Methods

n/a	Involved in the study
☒	☐ ChIP-seq
☒	☐ Flow cytometry
☒	☐ MRI-based neuroimaging

Clinical data

Policy information about clinical studies

All manuscripts should comply with the ICMJE guidelines for publication of clinical research and a completed CONSORT checklist must be included with all submissions.

Clinical trial registration

clinicaltrials.gov (NCT02977078), ISRCTN registry (ISRCTN90986892)

Study protocol

Full trial protocol unpublished. Available on request and from the University of Nottingham eTheses repository (http://eprints.nottingham.ac.uk/id/eprint/64632, Accessed 6 Jul 2022)

Data collection

The study was conducted at the Nottingham Respiratory Research Unit, Nottingham, UK and at some participants' primary care practices with overall participant enrollment taking place between December 2016 and December 2018. Interviews were conducted as part of the final study visit between July 2017 and June 2019.

Outcomes

N/A - report of qualitative data
